# Supplementary material for: NCI–Clinical Trial Accrual in a Community Network Affiliated with a Designated Cancer Center
Source: J Clin Med. 2020 Jun 24;9(6):1970. doi: 10.3390/jcm9061970 (PMC7355880; doi:10.3390/jcm9061970)
Supplement: Supplementary file 1 [file jcm-09-01970-s001.pdf]

Supplementary Materials:

Table S1. summary of phases and disease states of trials accrued to at community sites and City of Hope main campus.

Disease (IRB)	Disease State	Sponsor	Phase	Community	Main Campus
Palliative Care for phase I trials (13193)	Any	Peer-Reviewed IST	n/a		X
Solid Tumors (14023)	Metastatic, refractory	Institutional	I (first in human)		X
Breast – Triple Negative (14036)		Institutional	I/IB		X
Pancreatic (14122)	Metastatic or unresectable	Institutional	II	X	X
Nasopharyngeal (14258)	Definitive	Cooperative group	II/III	X	X
Prostate (15113)	mCRPC	Peer-Reviewed IST	II, randomized	X	X
Breast (15114)	Neoadjuvant, ER+	IST	II	X	X
NSCLC (15146)	?adjuvant	Cooperative Group	III		X
NSCLC (15147)	Adjuvant (marker identification and sequencing)	Cooperative Group	n/a	X	X
Geriatric (15161)	Any	IST	Pilot	X	X
Solid Tumors (15236)	Advanced	Cooperative group	I/II		X
Genitourinary (15256)	Advanced	NCI	I		X
Kidney (16357)	Adjuvant	Industry	III, randomized placebo controlled	X	X
Prostate (16358)	mCRPC, DNA repair mutation	Industry	II		X
Colorectal (16374)	Metastatic, refractory	IST	II		X
Prostate (16397)	mCRPC	Industry	III		X
NSCLC (16408)	IIIB/IV	Industry	I/II		X
Urothelial (16409)	Metastatic	NCI	II, randomized		X
Breast (16421)	Prevention	IST	II	X	X
Colorectal (16423)	Metastatic, MSS	IST	Pilot		X

All tumors (16441)	Receiving Radiation	Industry	Biomarker	X	X
Breast – triple negative (16451)	Adjuvant	Cooperative group	III, randomized	X	
Breast (17011)	Metastatic, previously treated	Industry	II		X
Solid Tumors (17027)	Any	Industry	I		X
NSCLC (17056)	Adjuvant	Cooperative Group	III	X	X
Kidney (17065)	Metastatic	Industry	II		X
Melanoma (17071)	Advanced	Industry	I		X
Ovarian (17095)	Advanced platinum refractory	Industry	I		X
Solid Tumor (17109)	Metastatic, Refractory	Indsutry	I		X
Prostate (17113)	mCRPC	Industry	III		X
Breast (17114)	Metastatic, previously treated	Industry	II		X
Solid Tumor (17144)	Metastatic	Industry	I		X
Bile duct (17148)	Metastatic, previously treated	Industry	III		X
Solid Tumor (17150)	Metastatic	Industry	I		X
NSCLC (17156)	Limited metastatic	Cooperative group	II/III	X	X
Gastric (17180)	Metastatic	Industry	III		X
Solid Tumor (17210)	Metastatic	Industry	I		X
Colorectal (17212)	Metastatic, previously treated	IST	I	X	X
Brain metastases (17237)	Metastatic Her2+	IST	I		X
RET-fusion solid tumors (17273)	Metastatic	Industry	I/II		X
Geriatric Solid Tumor (17314)	Not specified	Cooperative group	Feasibility		X
NSCLC (17318)	Metastatic, EGFR agent resistance	IST	I		X
Solid tumor (17332)	Metastatic	Industry	I		X
Colorectal / Carcinoid (?) (17352)	Metastatic	Industry	I		X
Pancreas (17363)	Metastatic	Peer reviewed (SU2C)	II		X
Geriatric Solid Tumor (17375)	Advanced	Peer reviewed	Observational	X	X

Melanoma (17378)	Metastatic, previously treated	Cooperative group	III	X	X
GIST, NEC (17379)	Advanced	Industry	I		X
NSCLC (17386)	Metastatic / advanced	Industry	II		X
Breast – Her2+ (17416)	Metastatic	Cooperative group	III	X	X
Hepatocellular (17420)	Advanced/ metastatic	Industry	III	X	X
Gastric (17450)	Metastatic	NCI	I/II	X	X
Geriatric Breast (17471)	Metastatic	IST	II	X	X
Urothelial (17472)	Metastatic, previously treated	IST	II		X
Cholangiocarcinoma (17493)	Metastatic / unresectable	NCI	II		X
Colorectal (17494)	Advanced	Industry	II		X
Solid Tumors (18056)	Metastatic, previously treated	Industry	I		X
Breast – Her2 neg (18090)	Adjuvant	Cooperative group	III	X	X
Solid Tumors (18100)	Metastatic	Industry	I/II		X
Biliary and pNET (18108)	Metastatic	Industry	I		X
Breast (18115)	Adjuvant	Cooperative group	III	X	X
Solid tumors (18137)	Metastatic	Industry	I		X
Prostate (18138)	mCRPC	IST	IB		X
Prostate (18140) supportive	Any, receiving ADT	IST	Pilot		X
Pancreas (18141)	Metastatic, previously treated	Industry	II/III		X
Prostate (18149)	Biochemically recurrent	Industry	III		X
Colorectal (18155)	Metastatic	Industry	I/II		X
NSCLC (18158)	Metastatic	Industry	I		X
Ovarian (18169)	Definitive (intraop imaging)	Industry	III		X
Pancreas 918227)	Metastatic	Industry	I/II		X
Breast (18321)	Adjuvant	Industry	III		X
Hepatocellular (18245)	Advanced	Industry	I/II		X
Colorectal (18280)	KRAS mutated, advanced	Industry	I/II		X

Oropharyngeal (18282)	HPV+, platinum refractory	Industry	II		X
Colorectal (18328)	Metastatic	Industry	I		X
Gastric (18329)	Metastatic, 1st line	Industry	II	X	X
Solid Tumors (18335)	Metastatic	Industry	I		X
Urothelial (18355)	Neoadjuvant	Cooperative group	II	X	X
Solid tumors (18411)	Metastatic, refractory to ICI	Industry	I/II		X
Prostate (18414)	Various (Imaging)	Industry	III		X
Cervical (18428)	Metastatic	Industry	I/II		X
Solid Tumors (18430)	Metastatic (imaging)	Industry	I/II		X
Breast – on aromatase inhib (18432)	Supportive	Cooperative group	Pilot	X	X
NSCLC (18465)	Metastatic	Industry	II	X	X
Hepatocellular (18473)	Advanced / Metastatic	Industry	III		X
Solid tumors – CCNE1 amp (18478)	Metastatic	NCI	II		X
Breast – triple negative (18496)	Metastatic	ISt	I/II		X
NSCLC (18512)	Metastatic	Industry	I/II		X
Kidney (18523)	Metastatic	ISt	Pilot randomized		X
Solid tumors (18525)	Metastatic	Industry	I		X
Solid tumor (18526)	Metastatic, previously treated	Industry	I		X
Breast – Her2+ (18556)	Adjuvant	IST	II		X
Biliary (19008)	Advanced, 1st line	Cooperative group	III	X	X
Solid tumors with ostomy (19147)	Supportive / telehealth	Peer reviewed	Pilot		X
